# Beyond the Eco-Label: How Credibility Shapes Sustainable Food Choice Online

**DOI:** 10.3390/foods15193562

**Published:** 2026-10-08

**Authors:** Yidong Liu, Bo Wan, Yansong Wen, Xiaozhe Li

**Affiliations:** 1 School of Design, Jianghan University, Wuhan 430056, China; vintonliu@hanyang.ac.kr (Y.L.); wanbo@jhun.edu.cn (B.W.); 2 Graduate School, Hanyang University, Seoul 04763, Republic of Korea; 3 City University of Hong Kong (Dongguan), Dongguan 523808, China; 72540797@cityu-dg.edu.cn

**Keywords:** sustainable food consumption, perceived diagnosticity, perceived value, food traceability, price sensitivity, online grocery retail

## Abstract

Eco-labels are important information cues for evaluating the environmental attributes of food products online, yet their effectiveness depends on whether consumers perceive these claims as credible and decision-relevant. This study examines how perceived food eco-label credibility shapes sustainable food purchase intention through two complementary cognitive mechanisms—perceived diagnosticity and perceived value—and whether these processes are contingent on food traceability and price sensitivity. Survey data were collected from 496 consumers of Hema Fresh, a major Chinese online grocery retail platform, and analyzed using structural equation modeling, bootstrap mediation analysis, and moderation analysis. Eco-label credibility positively influenced perceived diagnosticity (β = 0.415, *p* < 0.001) and perceived value (β = 0.417, *p* < 0.001), which subsequently increased sustainable food purchase intention (β = 0.372 and 0.295, respectively; both *p* < 0.001). Bootstrap results confirmed significant indirect effects through perceived diagnosticity (effect = 0.154, 95% CI [0.099, 0.215]) and perceived value (effect = 0.123, 95% CI [0.075, 0.179]). Food traceability strengthened the effects of eco-label credibility on both perceived diagnosticity (β = 0.215, *p* < 0.001) and perceived value (β = 0.103, *p* < 0.001). Price sensitivity weakened the perceived diagnosticity–purchase intention relationship, whereas its moderation of the perceived value–intention relationship was not significant. These findings reveal how credible eco-label information is translated into sustainable food choice through complementary information-diagnostic and value-evaluation processes, while identifying traceability and price sensitivity as distinct boundary conditions in online grocery retail.

## 1. Introduction

Environmental sustainability has become an increasingly important concern in contemporary food retail markets as climate change, ecological degradation, and resource scarcity continue to reshape consumer expectations and business practices. In response, firms have increasingly incorporated sustainability initiatives into their marketing strategies to encourage environmentally responsible consumption [1]. Among these initiatives, eco-labels have emerged as one of the most widely adopted mechanisms for communicating environmental attributes and sustainability-related information about food products, enabling consumers to identify products that align with their environmental values and purchasing goals [2].

Despite their widespread adoption, however, eco-labels do not always produce the intended behavioral outcomes, particularly in digital grocery retail environments. Unlike traditional offline shopping, online consumers cannot directly inspect food products or verify sustainability claims before purchase, making them more dependent on external informational cues when evaluating product quality and environmental performance [3,4]. Meanwhile, the rapid expansion of green marketing has intensified concerns regarding greenwashing and misleading environmental claims, increasing consumers’ skepticism toward sustainability information [5]. Under these conditions, the effectiveness of eco-labels depends not simply on their presence, but on whether consumers perceive them as credible signals capable of reducing uncertainty and supporting food product evaluation.

This perspective is consistent with signaling theory, which argues that credible signals help reduce information asymmetry when product attributes cannot be directly observed [6,7]. Within digital grocery retail environments, eco-labels function as signals of otherwise difficult-to-observe environmental attributes of food products. However, signaling theory also suggests that signals influence decision making only when they are regarded as sufficiently credible by receivers. Consequently, the central theoretical question is no longer simply whether eco-labels influence sustainable food consumption, but rather how eco-label credibility is cognitively translated into sustainable consumption intention. Although previous studies have consistently demonstrated positive relationships between eco-labels and sustainable purchasing [8,9], considerably less attention has been devoted to understanding the cognitive mechanisms through which eco-label credibility shapes consumers’ food choices in digital retail contexts.

Drawing on information processing theory, this study argues that consumers’ responses to credible eco-labels involve two complementary forms of cognitive evaluation rather than a single undifferentiated psychological response. One concerns whether sustainability-related information is sufficiently useful and applicable for distinguishing among food products and supporting purchase decisions. This information-evaluation process is reflected by perceived diagnosticity [10]. The other concerns whether the product and its sustainability attributes provide sufficient functional, environmental, and psychological benefits relative to the sacrifices associated with purchase, an evaluative process represented by perceived value [11]. Perceived diagnosticity therefore captures the decision usefulness of eco-label information, whereas perceived value captures consumers’ overall assessment of what they receive relative to what they give. Compared with constructs such as green trust, perceived risk, or general attitude, these two mechanisms capture distinct but complementary evaluations through which credible sustainability information can influence food choice. Although previous studies have examined perceived diagnosticity and perceived value separately [3,5], limited research has integrated them into a unified framework explaining how eco-label credibility influences sustainable consumption intention in digital grocery retail environments.

The effectiveness of these cognitive pathways may further depend on both contextual and individual boundary conditions. From a contextual perspective, traceability systems provide consumers with verifiable information regarding food origins, production processes, and supply-chain activities, thereby supporting the evaluation of sustainability claims [12]. Traceability may therefore strengthen the extent to which credible eco-label information is perceived as both diagnostically useful and valuable. From an individual perspective, price sensitivity reflects consumers’ tendency to emphasize economic sacrifice when making purchase decisions, potentially constraining the translation of favorable cognitive evaluations into sustainable purchasing intention [13]. Existing studies have investigated the influence of traceability on transparency and consumer trust, while separate streams of research have examined the role of price sensitivity in sustainable food purchasing. However, these research streams have largely developed independently, and relatively little attention has been devoted to understanding how informational transparency and economic considerations jointly shape consumers’ responses to credible eco-labels in digital grocery environments.

To address these gaps, this study develops a moderated mediation framework to examine how eco-label credibility influences sustainable food consumption intention in digital grocery retailing. Specifically, we propose that eco-label credibility enhances sustainable consumption intention through two parallel cognitive pathways—perceived diagnosticity and perceived value—while traceability strengthens the effects of eco-label credibility on these evaluations and price sensitivity constrains their translation into behavioral intention. Hema Fresh, a representative Chinese digital grocery retail platform that combines eco-labeled food products with established product traceability functions, provides an appropriate empirical context for examining these relationships.

This study makes three primary contributions. First, it extends eco-label and sustainable food consumption research by shifting attention from the mere presence of eco-labels to their credibility as a psychological foundation of consumer food choice. Second, drawing upon signaling theory and information processing theory, it develops and validates a dual-path cognitive framework that distinguishes the diagnostic usefulness of eco-label information from consumers’ evaluations of product value, thereby clarifying how credible sustainability cues influence sustainable food consumption intention. Third, by jointly examining traceability and price sensitivity, the study identifies contextual and individual boundary conditions that determine when eco-label credibility is more or less effective in shaping sustainable food choices within digital grocery retail environments.

Accordingly, this study addresses the following research questions:

RQ1: How does perceived eco-label credibility influence sustainable food consumption intention through perceived diagnosticity and perceived value?

RQ2: How does food traceability shape the effects of perceived eco-label credibility on perceived diagnosticity and perceived value?

RQ3: How does price sensitivity condition the effects of perceived diagnosticity and perceived value on sustainable food consumption intention?

Building on these research questions, the following section develops the theoretical framework and formulates the corresponding research hypotheses.

## 2. Literature Review and Hypotheses Development

### 2.1. Eco-Labels and Sustainable Consumption

In contemporary retail markets, consumers often find it difficult to directly evaluate product quality, environmental performance, and sustainability-related attributes before purchase, particularly in online shopping environments where physical product inspection is unavailable [14]. Under such conditions, eco-labels have gradually become important informational mechanisms for communicating environmental responsibility and sustainability commitments to consumers [15].

Previous studies suggest that eco-labels can facilitate sustainable purchasing decisions by simplifying complex environmental information and improving product transparency [16,17]. In digital retail contexts, consumers rely heavily on informational and visual cues during product evaluation, making eco-labels influential heuristic signals in shaping sustainability perceptions and consumption decisions [18]. Existing research further demonstrates that eco-labels can encourage environmentally responsible choices, improve green product preferences, and positively influence sustainable consumption behaviors across various product categories, including food, fashion, and online retail products [19,20,21].

Nevertheless, growing evidence indicates that the effectiveness of eco-labels depends heavily on whether consumers perceive them as trustworthy and reliable [5]. Consumers may become skeptical of environmental claims when sustainability information lacks transparency or when product attributes cannot be independently verified, especially in online retail environments where direct product examination is limited [3]. Consequently, eco-label credibility has emerged as a key factor influencing consumers’ responses to sustainability-related information [8]. Prior studies have shown that credible eco-labels can strengthen consumer trust, improve evaluations of sustainable products, and increase green purchase intentions [9,22]. Recent research also suggests that eco-labels may communicate broader value-related signals beyond environmental benefits, thereby shaping consumers’ overall consumption judgments [23].

Although research on eco-labels and sustainable consumption has expanded considerably, several important gaps remain. First, much of the existing literature focuses primarily on the direct relationship between eco-labels and purchase intention, while the underlying cognitive processes through which eco-label credibility influences sustainable consumption remain insufficiently explored. Second, limited attention has been given to online retail environments, where sustainable purchasing decisions are highly dependent on information transparency, informational cues, and product traceability [24]. Third, the boundary conditions that may weaken or strengthen the influence of eco-label credibility, particularly traceability and price sensitivity, remain underexplored. To address these gaps, we develop a moderated mediation framework to examine how eco-label credibility shapes sustainable consumption intention through perceived diagnosticity and perceived value in digital retail contexts.

### 2.2. Eco-Label Credibility and Cognitive Evaluation

Consumers purchasing eco-labeled products in online retail environments often face substantial information asymmetry because many sustainability attributes, such as environmental performance, ethical sourcing, and production practices, cannot be directly observed prior to purchase. Signaling theory provides a useful framework for explaining how consumers make judgments under such conditions. Spence [6] argues that when one party possesses information unavailable to another, credible signals help reduce information asymmetry by conveying otherwise unobservable product attributes. Building on this perspective, Connelly et al. [7] conceptualize signaling as a communication process in which signal receivers interpret the credibility of observable signals to infer hidden product characteristics. The effectiveness of a signal therefore depends not only on its existence but also on whether it is perceived as reliable, trustworthy, and difficult to imitate. Within sustainable consumption contexts, eco-labels represent important market signals that communicate environmental responsibility and sustainability performance. However, eco-labels can facilitate consumer decision-making only when they are considered credible. Once consumers perceive eco-labels as trustworthy, they are more likely to use the information conveyed by these labels to interpret product attributes and evaluate product benefits. Accordingly, eco-label credibility may influence sustainable purchasing decisions through two distinct cognitive evaluation processes: improving consumers’ perceptions of information diagnosticity and enhancing their perceptions of product value.

#### 2.2.1. Eco-Label Credibility and Perceived Diagnosticity

Perceived diagnosticity (PD) refers to consumers’ perceptions of the extent to which available information is useful, informative, and effective for evaluating product attributes and supporting purchase decisions [10]. In online retail environments, consumers are unable to physically inspect products and therefore depend heavily on external information when assessing quality, environmental performance, and sustainability characteristics [25]. Previous research has shown that various online informational cues, including website design, product descriptions, reviews, and interactive functions, significantly influence consumers’ perceptions of information diagnosticity in digital shopping environments [4,26]. Recent sustainable consumption research further suggests that when sustainability-related information is perceived as diagnostic, consumers become more confident in distinguishing genuinely sustainable products from misleading or exaggerated environmental claims, thereby facilitating environmentally responsible purchasing decisions [27,28].

From the perspective of signaling theory, eco-label credibility primarily influences consumers by improving the interpretability of sustainability-related information rather than directly changing value perceptions. Because many environmental attributes cannot be directly verified before purchase, consumers rely on credible signals to infer otherwise unobservable product characteristics [6]. Connelly et al. [7] further argue that receivers evaluate the credibility of market signals before using them to make judgments about hidden product attributes. Consequently, eco-labels are considered useful decision aids only when consumers believe that they accurately and reliably represent a product’s environmental performance.

This information interpretation process is further supported by signaling research on product quality. Kirmani and Rao [29] argue that credible and difficult-to-imitate signals reduce consumers’ uncertainty regarding unobservable product attributes, thereby improving the usefulness of available information in product evaluation. Similarly, Erdem and Swait [30] demonstrate that credible signals reduce information costs and increase consumers’ confidence in product assessment by facilitating more efficient information processing. Applied to eco-label contexts, credible environmental certifications enable consumers to interpret sustainability information more confidently, making eco-labels more informative and more useful for distinguishing environmentally responsible products from conventional alternatives. Consequently, consumers perceive sustainability-related information as having greater diagnostic value for purchase decisions. Based on this reasoning, the following hypothesis is proposed:

**H1.**  *Eco-label credibility positively influences perceived diagnosticity.*

#### 2.2.2. Eco-Label Credibility and Perceived Value

Perceived value (PV) refers to consumers’ overall evaluation of a product based on the trade-off between perceived benefits and perceived sacrifices [11]. Rather than reflecting the usefulness of information itself, perceived value represents consumers’ comprehensive assessment of the benefits associated with product consumption. In sustainable consumption contexts, these benefits extend beyond functional performance to include environmental, emotional, and social value derived from purchasing environmentally responsible products [31,32].

Although eco-labels provide sustainability-related information, their influence on perceived value arises through a different psychological mechanism from perceived diagnosticity. Once consumers accept eco-labels as credible signals, they are more likely to infer that eco-labeled products possess superior environmental quality, stronger ethical standards, and more reliable sustainability performance. Consequently, eco-label credibility not only facilitates information interpretation but also enhances consumers’ evaluations of the benefits associated with the product itself.

Previous research suggests that credible environmental certifications strengthen consumers’ perceptions of product quality, reliability, and environmental responsibility, thereby increasing overall product value [5,33]. Conversely, when eco-labels are perceived as ambiguous or unreliable, consumers become skeptical of environmental claims and discount the potential benefits associated with sustainable products [8]. Keller [34] further argues that credible market signals contribute to stronger consumer evaluations by reinforcing favorable product associations. Within online retail environments characterized by substantial information uncertainty, trustworthy eco-labels therefore function as credible indicators of both environmental quality and overall product benefits, enabling consumers to perceive eco-labeled products as more valuable, worthwhile, and deserving of purchase. Recent evidence also indicates that credible eco-labels strengthen consumers’ willingness to pay price premiums by reinforcing perceived environmental and quality-related value [35,36]. Accordingly, the following hypothesis is proposed:

**H2.**  *Eco-label credibility positively influences perceived value.*

### 2.3. Dual Cognitive Pathways to Sustainable Consumption Intention

Building on the signaling framework developed in the preceding section, this study proposes that eco-label credibility influences sustainable consumption intention (SCI) through two related but conceptually distinct cognitive processes. The first concerns information interpretation and is captured by perceived diagnosticity (PD): consumers assess whether eco-label information is sufficiently useful, relevant, and reliable to support product evaluation. The second concerns benefit evaluation and is represented by perceived value (PV): consumers judge whether the functional, environmental, ethical, and psychological benefits associated with an eco-labeled product justify the monetary and nonmonetary sacrifices required for purchase.

These two evaluations may be positively associated because useful information can facilitate subsequent value judgments. Nevertheless, they differ in both their object and function. PD evaluates the decision usefulness of sustainability information, whereas PV evaluates the overall worth of the product itself. Put differently, PD answers whether consumers can make an informed judgment based on the available information, while PV answers whether the product is worth choosing once its attributes have been assessed. This distinction provides the theoretical basis for treating PD and PV as complementary rather than interchangeable pathways to SCI.

#### 2.3.1. Perceived Diagnosticity and Sustainable Consumption Intention

SCI reflects consumers’ willingness to choose products and services that reduce environmental harm and support longer-term sustainability objectives [1]. Although such intentions may be shaped by environmental attitudes and norms, consumers must first be able to interpret the information on which sustainable choices are based. This requirement is especially important in digital retail environments, where products cannot be physically inspected and environmental attributes are often difficult to verify before purchase.

From an information-processing perspective, consumers selectively acquire, interpret, and integrate marketplace information in order to form product judgments [37]. PD captures the extent to which the available information is considered useful for performing this evaluative task [10]. When eco-label information is perceived as diagnostic, consumers can identify relevant environmental attributes more easily, compare alternatives more effectively, and reach more confident conclusions about which products are genuinely sustainable. By contrast, information that appears vague, irrelevant, or difficult to apply provides little support for choice, even when environmental issues are personally important.

Adaptive decision-making research further suggests that consumers adjust their decision strategies according to the quality of the information environment and the cognitive effort required to evaluate alternatives [38]. Diagnostic information reduces unnecessary search and simplifies attribute comparison, thereby increasing decision confidence and making a sustainable option easier to justify. This mechanism differs from value formation: PD does not imply that the product is necessarily more beneficial or worthwhile; rather, it enables consumers to make a clearer and more defensible judgment about its environmental credentials.

Prior studies conducted in online consumption settings similarly show that PD improves purchase decisions by lowering uncertainty and increasing confidence in product evaluation [25,28,39]. Within sustainable consumption contexts, this function is particularly important because consumers frequently face ambiguous claims and difficulty distinguishing genuinely sustainable products from less responsible alternatives [40]. When sustainability information is clear, actionable, and decision-relevant, consumers are more likely to translate environmental considerations into intended behavior [1,27]. Accordingly, this study proposes:

**H3.**  *Perceived diagnosticity positively influences sustainable consumption intention.*

#### 2.3.2. The Information-Interpretation Mechanism of Perceived Diagnosticity

The mediating role of PD reflects an information-interpretation mechanism rather than a general positive reaction to eco-labels. Credible eco-labels increase consumers’ confidence that the information displayed on the platform accurately represents otherwise difficult-to-observe environmental attributes. However, credibility alone does not directly ensure a sustainable choice. The information must also become usable within the consumer’s decision process.

When eco-label credibility (ELC) is high, consumers are more willing to treat sustainability claims as legitimate inputs for product evaluation [3]. Credibility therefore improves the likelihood that environmental information will be attended to, interpreted, and incorporated into comparison among alternatives. PD represents the point at which a credible signal becomes a practical decision aid: it captures whether consumers believe that the information actually helps them judge product sustainability, distinguish among competing options, and reduce ambiguity surrounding environmental claims.

This mechanism is consistent with information-processing research, which emphasizes that marketplace information affects choice only when consumers can transform it into evaluative judgments [37]. It is also consistent with adaptive decision theory, according to which consumers rely more heavily on information that improves judgment accuracy without imposing excessive cognitive effort [38]. Thus, the indirect effect through PD is not simply the statistical combination of ELC→PD and PD→SCI. It explains how a credible environmental signal becomes cognitively usable and, through greater decision confidence, supports the formation of sustainable consumption intention.

Empirical research in online reviews, livestream commerce, and sustainable product communication similarly indicates that PD can transmit the effect of informational cues to behavioral intention [27,39]. In the context of eco-labeled products, credible labels should therefore increase SCI to the extent that they help consumers form clearer and more informed judgments about environmental performance. Recent evidence further demonstrates that perceived diagnosticity serves as a cognitive mechanism through which information transparency influences consumers’ purchase intentions in online retail environments [41]. In the context of eco-labeled products, credible labels should therefore increase SCI to the extent that they help consumers form clearer and more informed judgments about environmental performance. Hence:

**H4.**  *Perceived diagnosticity mediates the relationship between eco-label credibility and sustainable consumption intention.*

#### 2.3.3. Perceived Value and Sustainable Consumption Intention

PV represents a different form of cognitive evaluation. Rather than assessing whether information is useful for making a judgment, PV concerns whether a product is worth acquiring after its expected benefits and required sacrifices have been considered. Zeithaml [11] defines value as an overall assessment based on what is received relative to what is given, while Sweeney and Soutar [31] show that this assessment can incorporate functional, emotional, and social benefits. In sustainable consumption, environmental contribution, ethical production, and psychological satisfaction may also become important components of the perceived return associated with purchase [32].

Woodruff [42] further conceptualizes customer value as an evaluation of product attributes, use consequences, and their ability to support consumers’ goals. This perspective is especially relevant to eco-labeled products because consumers must decide not only whether environmental claims are believable, but also whether the associated benefits are sufficiently meaningful to justify price premiums, additional effort, or other sacrifices. A consumer may therefore perceive eco-label information as highly diagnostic yet still decline to purchase if the product does not appear to offer adequate overall value.

PV influences SCI by increasing the attractiveness and perceived justification of sustainable alternatives. Products that are believed to deliver reliable quality, environmental benefits, ethical satisfaction, or alignment with personal values are more likely to be viewed as worthwhile choices. Prior research has consistently linked favorable value assessments with stronger intentions to purchase environmentally responsible products [32,43]. Similar relationships have been observed in sustainable fashion, green food, and other online consumption contexts [36,44,45].

Accordingly, PV affects SCI through a benefit–sacrifice judgment rather than through enhanced information clarity. When the expected environmental, functional, and psychological benefits of eco-labeled products outweigh their perceived costs, consumers have stronger reasons to choose them. Therefore:

**H5.**  *Perceived value positively influences sustainable consumption intention.*

#### 2.3.4. The Benefit-Evaluation Mechanism of Perceived Value

The mediating role of PV reflects a benefit-evaluation mechanism. Eco-label credibility can make the positive attributes associated with sustainable products appear more genuine, attainable, and meaningful. Credible labels signal that claimed environmental standards, ethical practices, and quality-related benefits are not merely promotional assertions but are more likely to represent actual product characteristics [8].

Once these benefits are accepted as credible, consumers can incorporate them into their overall assessment of what the product offers. This process differs from PD mediation. PD concerns whether the label information can support judgment; PV concerns whether the inferred product outcomes justify purchase. In other words, ELC raises PV by strengthening consumers’ beliefs that eco-labeled products provide real environmental, functional, and psychological returns, rather than simply by making the information easier to understand.

This reasoning is consistent with Woodruff’s [42] view that value arises from consumers’ evaluations of attributes and use consequences in relation to desired goals. In sustainable consumption, a credible eco-label may indicate that selecting the product contributes to environmental protection, ethical production, personal responsibility, or anticipated product quality. These outcomes increase the perceived worth of the product and provide a substantive motivational basis for sustainable consumption intention. Conversely, when an eco-label lacks credibility, consumers may discount the claimed benefits, weakening PV even if the information itself is visible and comprehensible.

Prior research supports the role of value as a mechanism through which sustainability cues influence behavioral intention [5,36,46]. Therefore, the indirect effect through PV captures how credible eco-labels transform inferred sustainability attributes into an overall judgment that the product is worth purchasing. Accordingly:

**H6.**  *Perceived value mediates the relationship between eco-label credibility and sustainable consumption intention.*

### 2.4. The Moderating Role of Traceability

#### 2.4.1. Traceability and Perceived Diagnosticity

Traceability (TR) describes the extent to which product-related information can be tracked, monitored, and verified across multiple stages of the supply chain, such as sourcing, manufacturing, processing, and distribution [12,47]. Within sustainable consumption contexts, traceability systems help improve information transparency and alleviate uncertainty surrounding environmental claims and product quality [48]. This function becomes particularly important in online retail settings, where consumers cannot physically inspect products and therefore rely heavily on external informational signals when evaluating sustainability-related attributes.

The existing literature suggests that transparent and verifiable traceability information can strengthen consumers’ perceptions of product authenticity, safety, and reliability by clarifying product origins and production procedures [49,50]. In eco-labeled consumption contexts, environmental claims tend to be viewed as more trustworthy when supporting information can be independently verified. Recent studies further indicate that traceability systems improve the credibility and transparency of sustainability communication, thereby enhancing the effectiveness of eco-labels [51,52]. Related evidence from certified sustainable products further shows that trust in eco-labels and perceived green supply-chain transparency jointly contribute to consumers’ sustainable purchase behavior, underscoring the importance of transparent supply-chain information in supporting the effectiveness of environmental certifications [53].

From an information-processing perspective, TR may reinforce the effect of eco-label credibility (ELC) on perceived diagnosticity (PD). Higher levels of traceability provide consumers with clearer and more verifiable sustainability-related information, enabling eco-labels to serve as more informative cues for evaluating environmental attributes. By contrast, limited or ambiguous traceability mechanisms may reduce consumers’ ability to verify sustainability claims, weakening the perceived usefulness of eco-label information. Therefore, stronger traceability systems are likely to enhance consumers’ perceptions of the diagnostic value associated with credible eco-labels.

Prior research also shows that transparent traceability mechanisms can reduce information asymmetry and uncertainty, making sustainability-related information appear more actionable and decision-relevant to consumers [54]. More directly, Yuan et al. [54] showed in the food consumption context that the informational properties of food traceability systems, including information quality, perceived reliability, and product diagnosticity, play important roles in shaping consumers’ evaluations and purchase decisions. This evidence further suggests that traceability can enhance the decision usefulness of product-related information by providing consumers with more reliable and verifiable information for product assessment. In digital retail environments, traceability may therefore amplify the positive influence of ELC on PD by improving consumers’ confidence in sustainability verification processes.

**H7a.**  *Food traceability positively moderates the relationship between eco-label credibility and perceived diagnosticity, such that the relationship becomes stronger under higher levels of food traceability.*

#### 2.4.2. Traceability and Perceived Value

Traceability systems not only increase information transparency but also strengthen consumers’ evaluations of product authenticity, reliability, and sustainability-related benefits [48]. Prior studies suggest that consumers tend to assign greater value to products whose information can be transparently tracked and verified across supply-chain processes [54]. In sustainable consumption settings, traceability mechanisms provide additional assurance regarding product origins, environmental performance, and manufacturing procedures, thereby enhancing perceived value (PV) toward eco-labeled products.

Previous research further demonstrates that traceability can positively shape value perceptions by reducing uncertainty and reinforcing trust in sustainability-related claims [12]. Especially in online retail contexts where direct product inspection is limited, transparent traceability information functions as an important informational signal that strengthens perceptions of environmental quality and ethical responsibility [51]. As a result, eco-labeled products supported by traceable sustainability information are more likely to be viewed as trustworthy, reliable, and worthwhile.

Moreover, traceability may strengthen the influence of ELC on PV because verifiable sustainability information improves consumers’ confidence in environmental claims. Signaling theory suggests that sustainability-related signals are perceived as more valuable when supported by transparent and credible evidence [52]. Under conditions of high traceability, consumers can more easily confirm whether eco-labeled products genuinely satisfy environmental standards, thereby reinforcing the value-enhancing effect of credible eco-labels. In contrast, weak traceability mechanisms may undermine confidence in sustainability claims and reduce the positive value associated with eco-label credibility.

Recent studies additionally indicate that traceability systems can enhance consumers’ willingness to pay premium prices for sustainable products by strengthening environmental and quality-related value perceptions [55]. Similarly, Anastasiadis et al. [56] emphasized that traceability contributes to greater transparency and trust within sustainable supply chains, which subsequently improves consumers’ value evaluations. This valuation effect is further supported by meta-analytic evidence showing that consumers generally assign positive economic value to food traceability and are willing to pay premiums for traceable food products [57]. Accordingly, in digital retail environments, TR is expected to strengthen the positive influence of ELC on PV.

**H7b.**  *Food traceability positively moderates the relationship between eco-label credibility and perceived value, such that the relationship becomes stronger under higher levels of food traceability.*

### 2.5. The Moderating Role of Price Sensitivity

Price sensitivity (PS) reflects the extent to which consumers emphasize monetary sacrifice when making purchase decisions and the degree to which price differences influence their product choices [58]. In sustainable consumption contexts, PS represents an important boundary condition because environmentally friendly products are frequently associated with price premiums relative to conventional alternatives [13]. However, price considerations do not necessarily intervene in all cognitive processes in the same manner. Their moderating role may depend on the stage of consumer decision-making at which they operate. Importantly, prior green consumption research has identified price sensitivity as a meaningful boundary condition in sustainable purchasing decisions. For example, Lavuri [59] empirically demonstrated the moderating role of price sensitivity in organic green purchasing, indicating that consumers’ responses to favorable sustainability-related evaluations can vary according to the importance they attach to price considerations.

In the present study, PS is expected to influence two different cognitive pathways leading to sustainable consumption intention (SCI). For consumers whose purchase decisions are primarily guided by the interpretation of sustainability information, price concerns may constrain the translation of diagnostic judgments into behavioral intention. For consumers whose decisions are based on an overall evaluation of product value, price sensitivity may further influence whether favorable value perceptions are ultimately converted into purchase intention, although perceived value itself already incorporates an assessment of benefits relative to sacrifices. Accordingly, PS is proposed as a boundary condition for both pathways, but through different psychological mechanisms.

#### 2.5.1. Price Sensitivity and the Relationship Between Perceived Diagnosticity and SCI

Perceived diagnosticity (PD) enhances consumers’ ability to evaluate product attributes and reduces uncertainty associated with sustainable purchasing decisions [25,39]. When sustainability-related information is considered highly diagnostic, consumers are more confident in distinguishing genuinely sustainable products from conventional alternatives, thereby increasing their willingness to engage in sustainable consumption.

Nevertheless, forming a confident judgment does not necessarily guarantee purchase behavior. Consumers with high PS often place greater emphasis on financial sacrifice than on informational certainty when making purchase decisions [60]. Although diagnostic information enables them to identify environmentally preferable products, high anticipated costs may still discourage them from acting on those judgments. In this sense, PS functions as an economic constraint that limits the conversion of informed product evaluations into sustainable purchasing intentions.

Previous studies similarly suggest that economic concerns may weaken the positive influence of sustainability-related evaluations on green purchasing behavior [59,61,62]. Consumers with relatively low PS, by contrast, are less constrained by monetary considerations and are therefore more likely to translate diagnostic sustainability information into actual purchase intention. Accordingly, the positive influence of PD on SCI is expected to be stronger among consumers with lower levels of PS.

**H8a.**  *Price sensitivity negatively moderates the relationship between perceived diagnosticity and sustainable consumption intention, such that the relationship is stronger when price sensitivity is low.*

#### 2.5.2. Price Sensitivity and the Relationship Between Perceived Value and SCI

Perceived value (PV) represents consumers’ overall evaluation of whether the benefits provided by a product outweigh the associated monetary and nonmonetary sacrifices [11]. In sustainable consumption contexts, this evaluation incorporates not only functional performance but also environmental, ethical, and psychological benefits [31]. Consequently, PV already reflects a comprehensive assessment in which price constitutes one important component of consumers’ overall value judgment.

Although price considerations are embedded within value evaluation, individual differences in PS may still influence the extent to which favorable value perceptions are translated into behavioral intention. Consumers who are highly sensitive to price may attach greater weight to economic sacrifice even after forming positive value evaluations, whereas consumers with lower PS may place relatively greater emphasis on environmental and ethical benefits when making final purchase decisions [13].

Existing research has shown that PS can reduce consumers’ willingness to purchase sustainable products despite favorable evaluations of sustainability-related attributes [63,64]. Similarly, consumers may perceive eco-labeled products as valuable while remaining cautious toward purchasing decisions if anticipated price premiums exceed their acceptable spending threshold [65,66]. Recent green consumption research has likewise examined price sensitivity as a boundary condition in the relationship between consumers’ favorable product evaluations, including perceived value, and green purchase intention [67]. This evidence reinforces the view that favorable value judgments do not necessarily translate uniformly into sustainable purchase intentions across consumers with different levels of price sensitivity. Therefore, although PV already incorporates consumers’ evaluation of costs and benefits, PS may provide an additional boundary condition affecting whether these favorable value perceptions are ultimately translated into sustainable consumption intention. Accordingly, this study proposes that the positive relationship between PV and SCI will be weaker among consumers with higher PS.

**H8b.**  *Price sensitivity negatively moderates the relationship between perceived value and sustainable consumption intention, such that the relationship is stronger when price sensitivity is low.*

### 2.6. Conceptual Model

**Figure 1:**
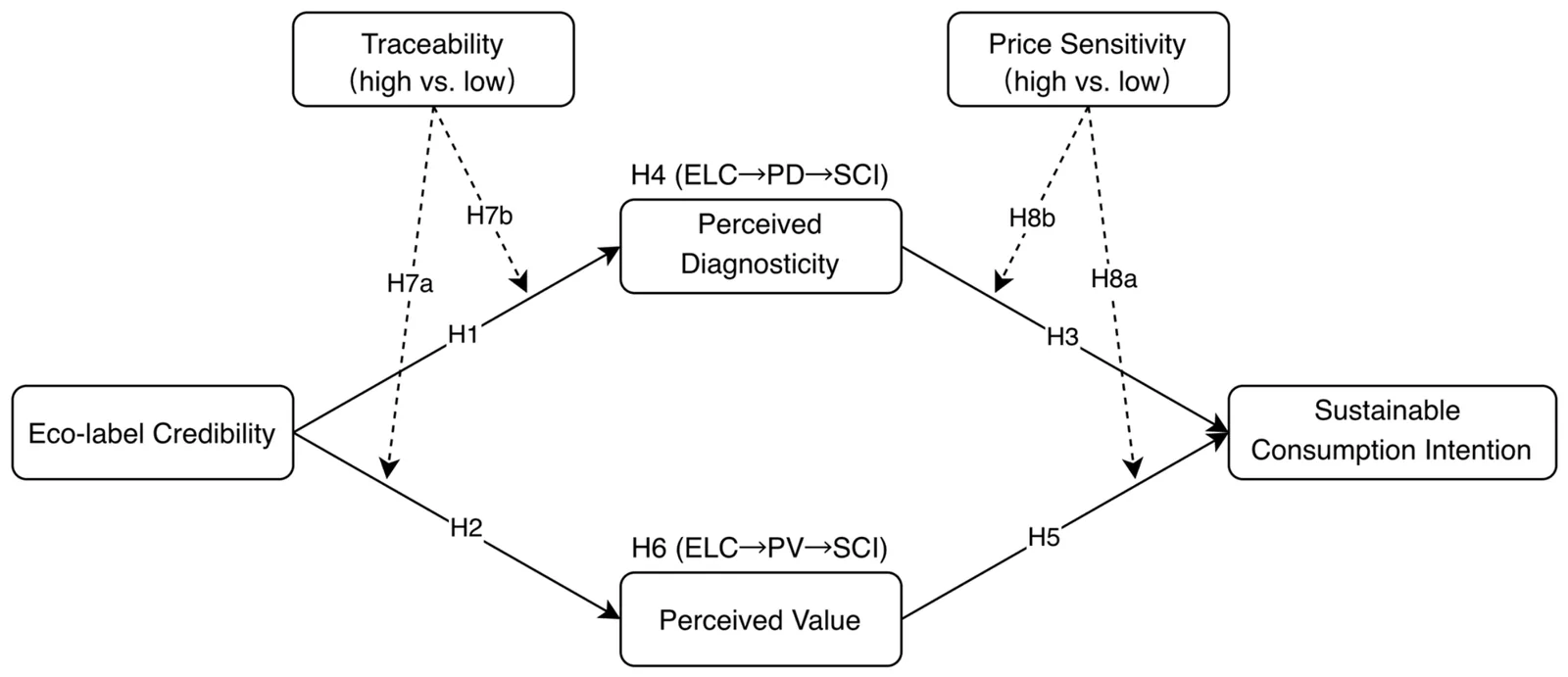
Research Model.

Integrating the above hypotheses, this study developed the research model shown in Figure 1.

## 3. Data and Methodologies Employed

### 3.1. Study Case

This study selected Hema Fresh (Hema Xiansheng), one of China’s leading digital retail platforms, as the empirical research context. Hema Fresh integrates online shopping, smart retail technologies, and supply chain management into a unified digital consumption platform, providing consumers with extensive access to eco-labeled and sustainability-related products. In recent years, environmentally friendly consumption has become increasingly prominent within Chinese digital retail markets, and Hema Fresh has actively introduced various sustainability-related labels and traceability systems for fresh food, organic products, and environmentally certified goods.

The selection of Hema Fresh as the study case is appropriate for several reasons. First, the platform provides a highly representative online retail environment in which consumers rely heavily on digital information cues, eco-labels, and traceability information when making purchasing decisions. Unlike traditional offline shopping contexts, consumers on digital retail platforms cannot directly inspect products, making sustainability-related informational signals particularly important for evaluating product quality and environmental attributes.

Second, Hema Fresh has established relatively comprehensive product traceability mechanisms that allow consumers to access information regarding product origins, production processes, and supply chain transparency through the platform interface. Such characteristics closely align with the theoretical focus of this study on eco-label credibility, perceived diagnosticity, perceived value, and traceability. The platform therefore provides an appropriate context for examining how sustainability-related informational cues influence consumers’ sustainable consumption intentions in digital retail environments.

Finally, eco-labeled products on Hema Fresh cover multiple categories, including organic food, environmentally certified agricultural products, and low-carbon products, enabling consumers to encounter diverse forms of sustainability information during the shopping process. This makes the platform particularly suitable for investigating the cognitive and behavioral mechanisms underlying sustainable consumption in online retail contexts.

### 3.2. Questionnaire

The survey was administered in Chinese because the target respondents were Chinese consumers. All measurement items were adapted from well-established English-language scales reported in previous studies (see Appendix A). Because the survey was administered exclusively in Chinese, the original English measurement items were translated into Chinese and then independently back-translated into English by bilingual researchers. The back-translated items were compared with the original English items to assess conceptual equivalence and linguistic accuracy; the English back-translated version was used solely for this verification purpose and was not administered to respondents. Any discrepancies identified during this process were resolved through discussion until consensus was reached. The final questionnaire was administered in Chinese to eligible users with prior experience using the Hema Fresh online shopping platform.

Before the formal survey, a pilot study involving 54 respondents was conducted to assess the clarity and preliminary psychometric properties of the measurement instrument. The pilot results demonstrated satisfactory internal consistency, with Cronbach’s α values of 0.832 for Eco-label Credibility, 0.885 for Perceived Diagnosticity, 0.867 for Perceived Value, 0.916 for Traceability, 0.888 for Price Sensitivity, and 0.925 for Sustainable Consumption Intention. All values exceeded the commonly recommended threshold of 0.70. In addition, the Kaiser–Meyer–Olkin (KMO) measure was 0.741, indicating adequate sampling adequacy for preliminary factor assessment. These results provided initial evidence of the reliability and suitability of the measurement instrument for the formal survey. Prior to completing the questionnaire, all participants were informed of the research purpose and assured that their responses would remain anonymous and be used solely for academic research. Participation was entirely voluntary, and respondents could proceed to the questionnaire only after providing informed consent.

The target population consisted of consumers with prior experience using the Hema Fresh online shopping application. Data were collected between 5 May and 20 May 2026, through the Chinese online survey platform Wenjuanxing. Respondents were recruited primarily via the Wenjuanxing platform, supplemented by convenience recruitment through the research team’s social media networks (e.g., WeChat and QQ), where invitations were distributed to potential participants who met the screening criteria. The primary behavioral eligibility criterion was prior and repeated use of the Hema Fresh online shopping platform. Before entering the formal questionnaire, respondents were required to confirm that they had used the platform at least five times during the previous six months. The target population was therefore not intended to represent the general Chinese population or Chinese food shoppers as a whole, but rather consumers with recent and repeated experience using Hema Fresh’s online shopping platform. The six-month period was used to ensure recency of experience, while the minimum-use requirement was intended to exclude occasional or inexperienced users and ensure sufficient familiarity with the platform’s product information environment, including eco-label and traceability information. Importantly, respondents were not required to be regular purchasers of eco-labeled or sustainable food products, because the study aimed to examine consumers’ perceptions and behavioral intentions rather than restricting the sample to consumers who had already adopted sustainable purchasing practices.

The questionnaire consisted of three main sections. The first section introduced the study and obtained respondents’ informed consent, followed by a screening question concerning their prior use of the Hema Fresh online shopping platform. The second section contained the 29 measurement items assessing the six focal constructs: Eco-label Credibility (ELC), Perceived Diagnosticity (PD), Perceived Value (PV), Food Traceability (TR), Price Sensitivity (PS), and Sustainable Consumption Intention (SCI). The final section collected respondents’ sociodemographic information, including gender, age, education, and monthly income. The complete measurement instrument is provided in Appendix A. All items were measured using a seven-point Likert scale ranging from 1 (strongly disagree) to 7 (strongly agree). This response format was adopted because seven-point scales provide sufficient response differentiation and have demonstrated favorable psychometric performance in terms of reliability, validity, and discriminating power [68]. A total of 559 questionnaires were collected. Responses were excluded if the questionnaire was completed in less than 60 s or if the respondent selected the same response option for all substantive questionnaire items, indicating insufficient engagement with the survey. Based on these criteria, 63 responses were excluded, leaving 496 valid questionnaires for subsequent analysis and yielding an effective response rate of 88.7%. The adequacy of the final sample was evaluated in relation to the analytical requirements of structural equation modeling rather than the size of the general Chinese population. SEM sample-size requirements depend on model characteristics, including the number of indicators and latent factors, factor loadings, path coefficients, and missing data patterns. Monte Carlo evidence reported by Wolf et al. [69] showed that required sample sizes across common SEM specifications varied substantially, ranging from approximately 30 to 460 observations. Accordingly, the final sample of 496 respondents was considered adequate for estimating the proposed SEM, while no claim of national representativeness is made.

Because all data were collected using a single questionnaire, the potential for common method bias (CMB) was assessed using Harman’s single-factor test. An unrotated exploratory factor analysis was conducted on all measurement items. The results extracted six factors with eigenvalues greater than 1, and the first factor accounted for 33.008% of the total variance, which is below the recommended threshold of 40%. These results indicate that common method bias is unlikely to pose a serious concern in the present study.

## 4. Results

### 4.1. Sample Profile

A summary of participants’ demographic characteristics is presented in Table 1.

As shown in Table 1, female respondents accounted for a slightly larger proportion of the sample (55.8%). The age distribution was relatively balanced, with respondents aged 36–45 representing the largest group (26.2%). Most participants (80.8%) held a college or university degree, indicating a relatively well-educated sample. In terms of monthly income, respondents generally belonged to the middle- and upper-income segments, with 32.7% reporting monthly incomes between RMB 8001 and 10,000 and nearly 30% earning more than RMB 10,000 per month. For contextual comparison, official statistics from the National Bureau of Statistics of China indicate that the average annual wages of employees in urban private and non-private units were RMB 71,590 and RMB 129,441, respectively, in 2025, equivalent to approximately RMB 5966 and RMB 10,787 per month. These national benchmarks provide useful context for interpreting the income distribution of the present sample. It should be noted that China does not apply a single nationwide minimum wage, as minimum wage standards vary across provincial-level regions.

This demographic profile is broadly consistent with Hema Fresh’s positioning as a premium retail platform serving consumers with relatively strong purchasing power. Accordingly, the findings should be interpreted primarily within this consumer segment, and caution should be exercised when generalizing the results to populations with substantially different demographic or socioeconomic characteristics.

### 4.2. Analytical Criteria

Before presenting the empirical results, the measurement and structural models were evaluated using established criteria. Measurement quality was assessed using standardized factor loadings, Cronbach’s α, composite reliability (CR), average variance extracted (AVE), the Fornell–Larcker criterion, and the heterotrait–monotrait ratio (HTMT). Standardized factor loadings of at least 0.50, Cronbach’s α and CR values of at least 0.70, AVE values of at least 0.50, and HTMT values below 0.85 were adopted as the corresponding evaluation criteria [70,71]. Overall model fit was assessed using χ^2^/df, RMSEA, IFI, TLI, CFI, GFI, and NFI, with χ^2^/df below 3.00, RMSEA below 0.08, and incremental and comparative fit indices of approximately 0.90 or above indicating acceptable model fit.

### 4.3. Confirmatory Factor Analysis

The convergent validity of the scales is as follow.

Table 2 reports the convergent validity results of the measurement model. The results indicate that all indicators satisfied the recommended criteria (Factor Loading > 0.5; CR > 0.7; AVE > 0.5), demonstrating satisfactory convergent validity [70]. Based on these results, discriminant validity was further examined, as presented in Table 3.

As shown in Table 3, the correlations among latent constructs were all lower than the square roots of the corresponding AVE values, indicating satisfactory discriminant validity for all constructs in accordance with the recommended criterion [71].

Table 3 also reports the skewness and kurtosis statistics for all constructs. The results indicate that the data approximate a normal distribution, satisfying the normality assumption required for structural equation modeling. Accordingly, the use of maximum likelihood estimation (MLE) in the confirmatory factor analysis (CFA) and structural equation modeling (SEM) was considered appropriate.

To further assess discriminant validity, the HTMT was also examined, as recommended in recent methodological research. As shown in Table 4, all HTMT values were below the conservative threshold of 0.85, providing additional evidence that the constructs are empirically distinct. These findings further confirm the satisfactory discriminant validity of the measurement model. Taken together, these results indicate that the measurement model demonstrates satisfactory reliability, convergent validity, and discriminant validity.

### 4.4. Structural Equation Modeling

**Figure 2:**
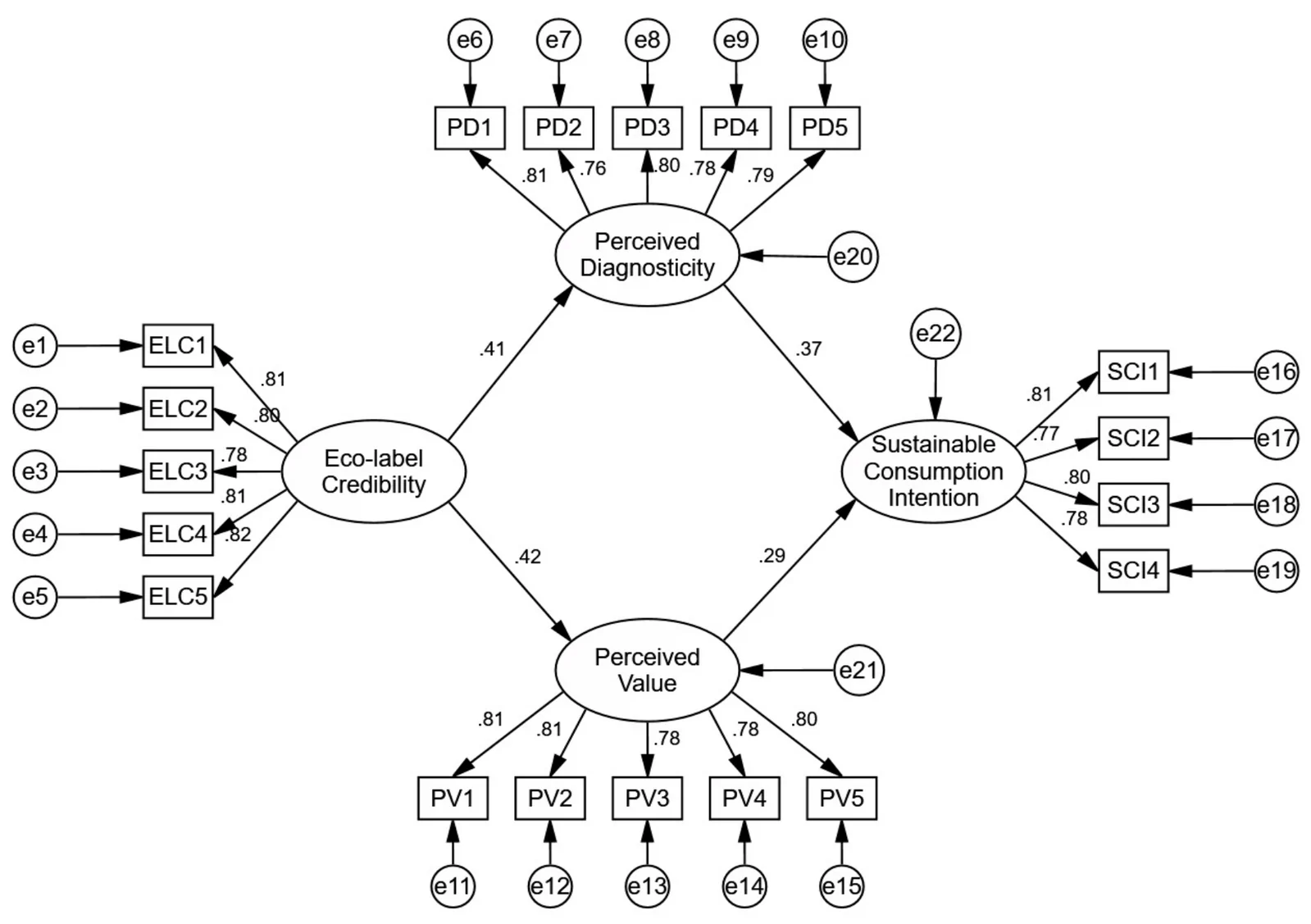
Results of SEM.

The above results indicate that the reliability and validity of the scales met the recommended standards. Based on these findings, structural equation modeling was further conducted using AMOS 24.0, with the results presented in Figure 2 and Table 5.

As shown in Table 5, all model fit indices met the recommended criteria. Based on this, the structural paths were further tested, and the results are presented in Table 6.

### 4.5. Mediation Effect

Bootstrapping analysis was conducted to examine the mediating effects within the proposed model. The results are reported in Table 7.

As presented in Table 7, the indirect effects of ELC on SCI through PD and PV were both significant (estimate = 0.154, SE = 0.030, 95% CI: [0.099, 0.215]; estimate = 0.123, SE = 0.026, 95% CI: [0.075, 0.179]), thereby supporting H4 and H6.

### 4.6. Moderating Effect

Moderation effects were examined using regression analysis by testing the significance of the interaction term between the independent variable and the continuous moderator. A significant interaction term indicates the presence of a moderation effect. To facilitate interpretation, significant interactions were further illustrated using simple slope plots. Specifically, the moderator was plotted at one standard deviation above and below its mean to represent high and low levels, respectively, allowing the relationship between the independent and dependent variables to be compared across different levels of the moderator. It should be noted that these high- and low-level groupings were used solely for visualization purposes; all statistical tests of moderation were conducted using the continuous interaction term.

**Figure 3:**
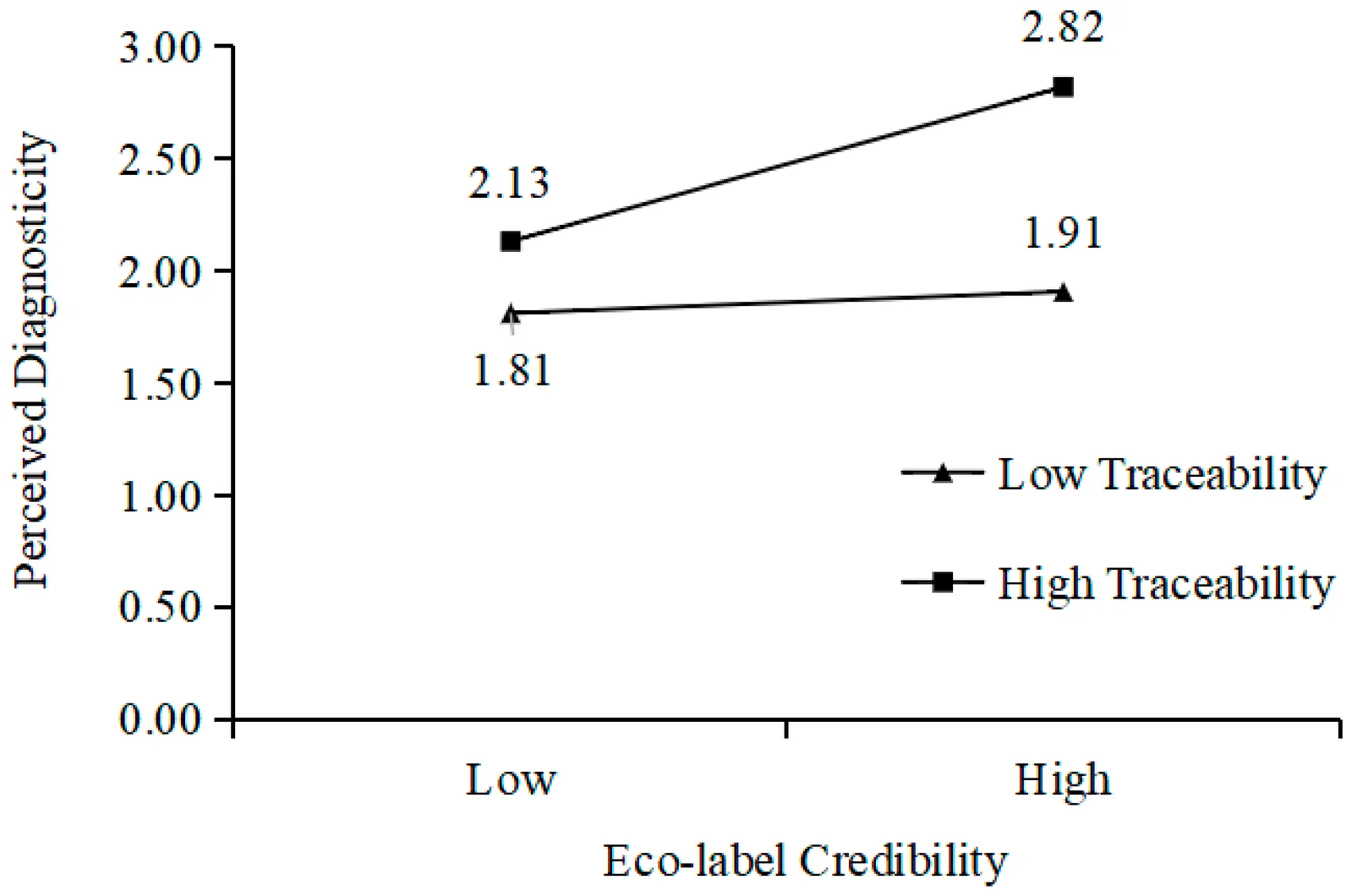
Moderating role of TR with ELC and PD.

**Figure 4:**
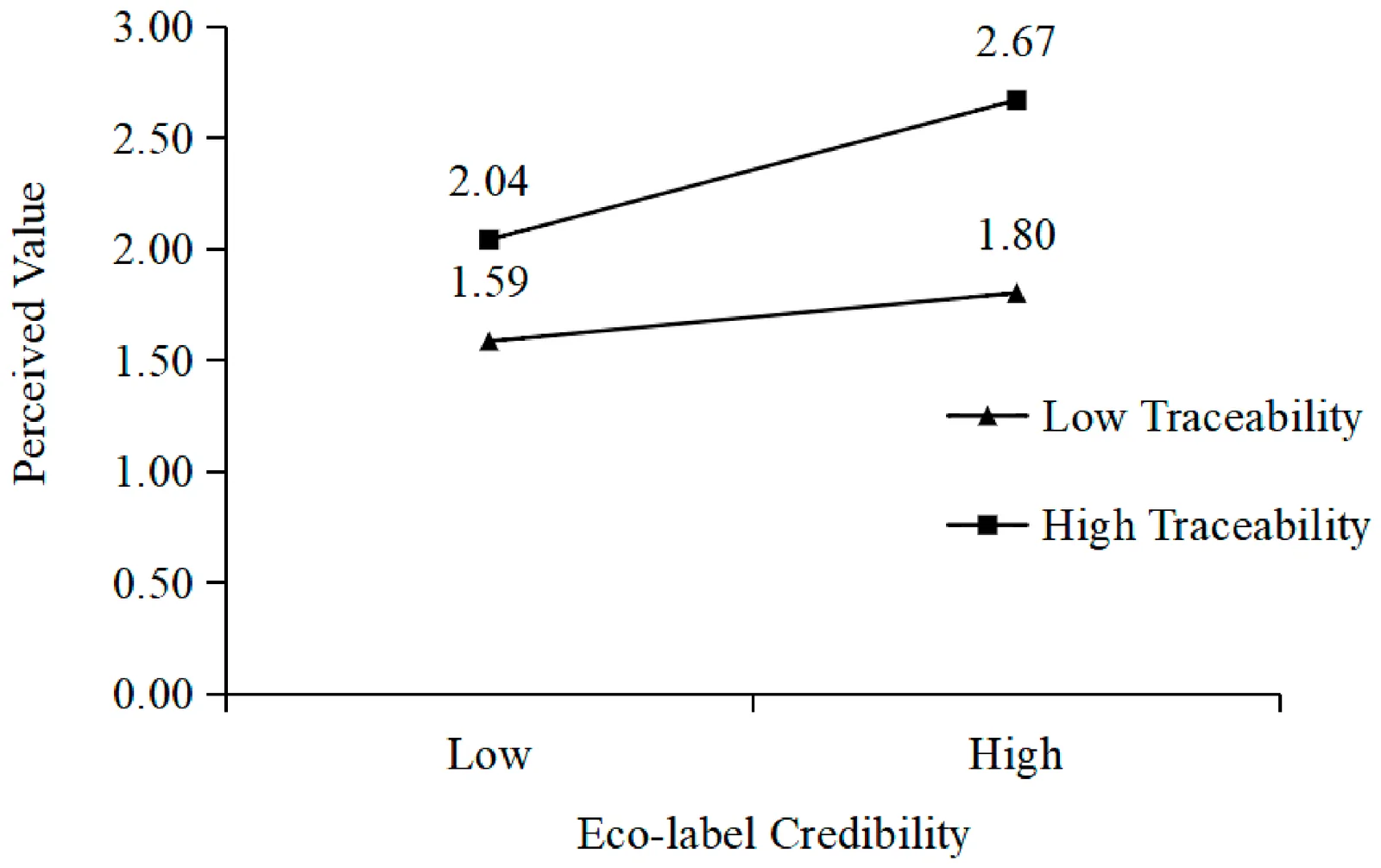
Moderating role of TR with ELC and PV.

First, the moderating effect of TR on the relationships between ELC and PD/PV was tested. The findings indicate that TR positively moderates the effects of ELC on both PD and PV (β = 0.215, SE = 0.027, *p* < 0.001; β = 0.103, SE = 0.028, *p* < 0.001). The interaction effects are illustrated in Figure 3 and Figure 4, supporting H7a and H7b.

**Figure 5:**
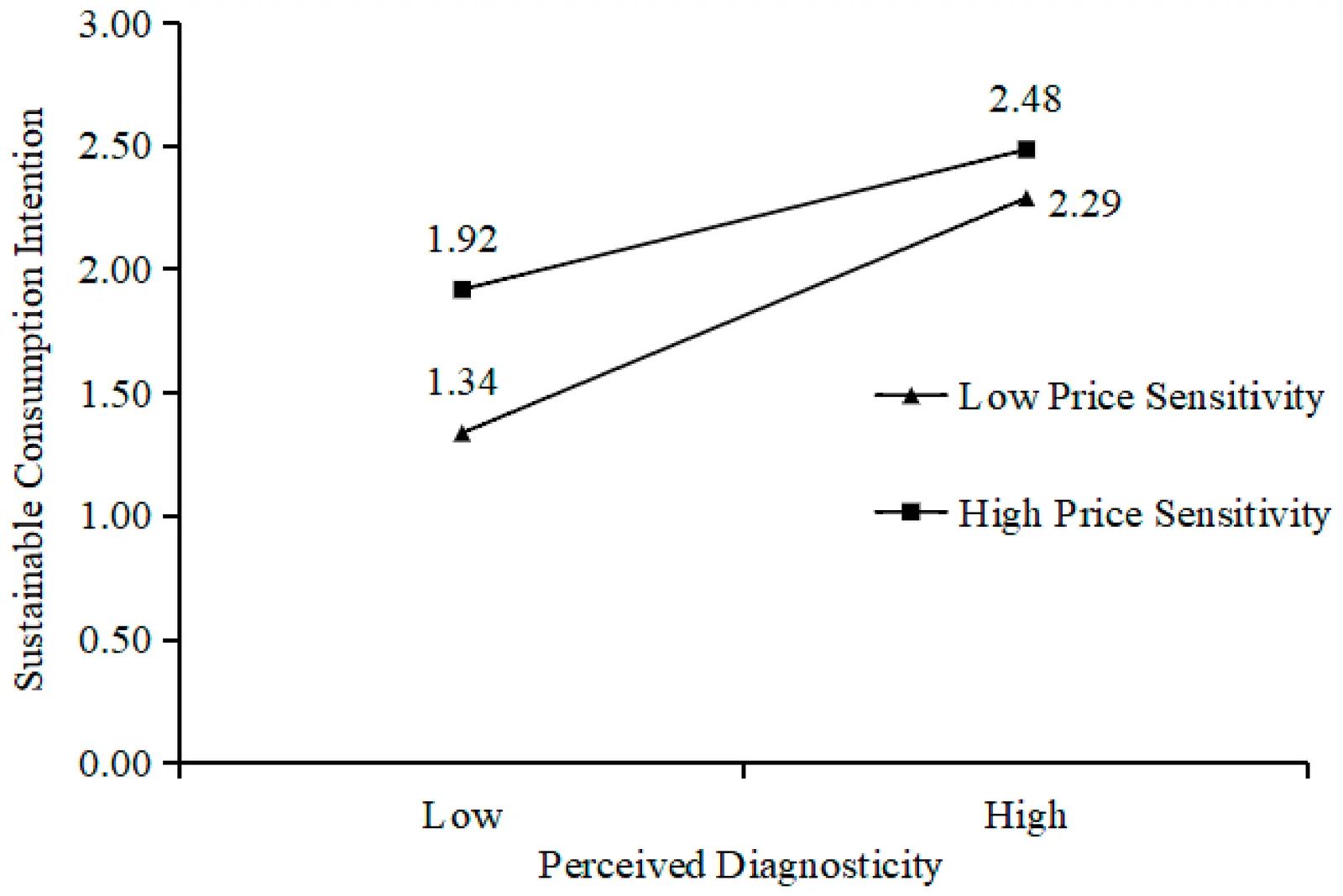
Moderating role of PS with PD and SCI.

In addition, the moderating role of PS in the relationships between PD/PV and SCI was examined. The results reveal that PS negatively moderates the relationship between PD and SCI (β = −0.125, SE = 0.031, *p* < 0.01), as illustrated in Figure 5, thereby supporting H8a. However, the interaction effect between PV and SCI was not statistically significant (β = −0.080, SE = 0.032, *p* > 0.05). Therefore, H8b was not supported.

## 5. Conclusions and Discussion

This study examined how eco-label credibility shapes sustainable food consumption intention in digital grocery retail through two complementary cognitive mechanisms—perceived diagnosticity and perceived value—and further investigated the boundary roles of food traceability and price sensitivity. The findings provide consistent support for the proposed dual-path framework. Eco-label credibility positively influenced both perceived diagnosticity and perceived value, which in turn enhanced sustainable consumption intention. Both variables significantly mediated the effect of eco-label credibility on sustainable consumption intention. Food traceability strengthened the effects of eco-label credibility on both perceived diagnosticity and perceived value. By contrast, price sensitivity exhibited an asymmetric moderating pattern: it weakened the relationship between perceived diagnosticity and sustainable consumption intention, whereas its moderating effect on the perceived value–intention relationship was not statistically significant. Taken together, these findings show that credible eco-labels influence sustainable food choice not through a single generalized positive response, but through distinct information-interpretation and benefit-evaluation processes whose effectiveness depends on different informational and economic conditions.

First, the positive effects of eco-label credibility on perceived diagnosticity and perceived value clarify how credibility enables sustainability information to become behaviorally meaningful in online grocery environments. Previous eco-label research has established that credible environmental labels can reduce uncertainty, strengthen trust in environmental claims, and improve evaluations of sustainable products. The present findings are consistent with this general literature but extend it by distinguishing two cognitive consequences of credibility. On the one hand, credible eco-labels make sustainability information more useful for evaluating and differentiating food products, thereby increasing perceived diagnosticity. This finding corresponds with research showing that reliable and transparent information enhances consumers’ ability to evaluate products in digital environments and that perceived diagnosticity provides an important cognitive link between informational cues and behavioral responses. Recent evidence similarly identifies perceived diagnosticity as a mechanism through which transparent online information can influence purchase intention [41]. On the other hand, eco-label credibility also increases perceived value by strengthening consumers’ confidence that the environmental, ethical, and quality-related benefits associated with eco-labeled products are genuine rather than merely promotional claims. This is consistent with previous sustainable consumption research linking credible environmental information with stronger product evaluations and willingness to accept sustainability-related benefits and price premiums.

Importantly, the significant mediating effects of perceived diagnosticity and perceived value show that these mechanisms are complementary rather than interchangeable. Previous studies have often examined the informational usefulness of sustainability cues and consumers’ value assessments in separate research streams. By incorporating both mechanisms within the same framework, the present study demonstrates that credible eco-labels can facilitate sustainable consumption through two conceptually different forms of evaluation. Perceived diagnosticity concerns whether sustainability information is sufficiently useful and actionable to support product judgment, whereas perceived value concerns whether the resulting environmental, functional, and psychological benefits make the product worth choosing. The significant indirect effects through both mechanisms therefore extend prior research by showing that credibility must not only make environmental information believable, but also enable consumers to use that information in decision making and recognize meaningful benefits from the sustainable product. This distinction is particularly relevant in digital grocery retail, where consumers cannot directly inspect many environmental attributes and must rely heavily on platform-mediated information when forming product judgments.

Second, food traceability significantly strengthened the relationships between eco-label credibility and both perceived diagnosticity and perceived value. These results are consistent with previous research emphasizing the informational and evaluative benefits of traceability, while also extending that literature in an important way. Yuan et al. [54], for example, showed that food traceability systems can improve consumers’ evaluations and purchase decisions through informational properties such as information quality, reliability, and product diagnosticity. Meta-analytic evidence further indicates that consumers generally assign positive economic value to food traceability and are willing to pay premiums for traceable food products [57]. Similarly, research on certified sustainable products has demonstrated that eco-label trust and supply-chain transparency jointly contribute to sustainable purchasing behavior [53]. Whereas these studies primarily establish the direct informational, trust-related, or value-related benefits of traceability, the present findings demonstrate that traceability can also alter the effectiveness of eco-label credibility itself.

More specifically, when food traceability is high, consumers have access to more verifiable information concerning product origins, production processes, and supply-chain activities. Under these conditions, a credible eco-label is supported by additional evidence that enables consumers both to interpret sustainability claims more confidently and to attribute greater value to the associated product benefits. When traceability is limited, by contrast, even a credible eco-label provides fewer opportunities for independent verification. Traceability therefore appears to function not merely as another favorable product attribute but as a verification mechanism that reinforces the informational value of credible environmental signals. This finding extends signaling-based explanations of sustainable consumption by suggesting that the effectiveness of one sustainability signal can depend on the presence of complementary information that supports its verification. In digital food retailing, credibility and traceability should therefore not be understood as isolated informational cues; rather, their combination creates a stronger information environment in which sustainability claims become simultaneously more decision-useful and more valuable.

Third, the findings concerning price sensitivity provide a more differentiated picture of the economic constraints surrounding sustainable food choice. Price sensitivity significantly weakened the positive relationship between perceived diagnosticity and sustainable consumption intention, supporting the proposition that being able to identify and evaluate a sustainable option does not necessarily ensure willingness to choose it. This result is consistent with previous green consumption research identifying price sensitivity as an important boundary condition in environmentally responsible purchasing [59]. Consumers with high price sensitivity may recognize that eco-label information is reliable, useful, and sufficient for distinguishing sustainable products, yet still hesitate to translate this judgment into purchase intention when monetary sacrifice receives greater weight in the final decision. The finding therefore extends previous research by locating the constraining role of price sensitivity at a specific stage of the decision process: economic concerns can interrupt the translation of diagnostic sustainability judgments into behavioral intention.

However, price sensitivity did not significantly moderate the relationship between perceived value and sustainable consumption intention. This finding is noteworthy because previous research has suggested that price sensitivity can condition the relationship between favorable product evaluations, including perceived value, and green purchase intention (e.g., Riyaz et al. [67]). The present result therefore indicates that the constraining effect of price sensitivity may not operate uniformly across different cognitive pathways. One plausible explanation concerns the conceptual nature of perceived value itself. Perceived value already represents an overall assessment of what consumers receive relative to what they sacrifice, meaning that monetary cost is incorporated, at least partly, into the formation of the value judgment. Once consumers have concluded that the environmental, functional, and psychological benefits of an eco-labeled product justify the associated sacrifices, individual differences in price sensitivity may provide relatively little additional explanatory power in determining whether this favorable value assessment translates into sustainable consumption intention. By contrast, perceived diagnosticity primarily reflects the usefulness of information rather than a benefit–cost evaluation, leaving greater scope for price concerns to intervene subsequently.

The contrast between these two moderating results provides an important refinement to the general assumption that price concerns uniformly suppress sustainable purchasing. Rather, economic constraints appear to depend on the cognitive route through which consumers arrive at a sustainable choice. Price sensitivity can weaken an information-based route because recognizing which option is environmentally preferable does not resolve whether the consumer is willing to bear its financial consequences. A favorable value judgment, however, already implies that the consumer has evaluated relevant benefits against sacrifices. This asymmetric pattern helps explain why the same economic characteristic can exert different effects across the two pathways and reinforces the conceptual distinction between perceived diagnosticity and perceived value established in the proposed model.

Overall, the findings refine understanding of sustainable food choice in digital retail environments by showing that credible sustainability information becomes effective through an interaction between signal credibility, cognitive evaluation, verification conditions, and economic considerations. Prior studies have separately emphasized eco-label credibility, traceability, perceived value, information diagnosticity, or price-related barriers. The present study connects these research streams by demonstrating that credible eco-labels generate sustainable consumption intention through both information-diagnostic and value-evaluation mechanisms, that traceability reinforces both mechanisms at the stage of credibility interpretation, and that price sensitivity selectively constrains the subsequent translation of diagnostic judgments into behavioral intention. Sustainable food choice online should therefore be understood not simply as a response to the presence of environmental information, but as a multi-stage evaluative process in which consumers determine whether sustainability information is credible, whether it is useful for decision making, whether the associated product is worth choosing, and whether economic considerations ultimately permit favorable evaluations to translate into intended behavior.

## 6. Implications

### 6.1. Theoretical Implications

First, this research advances the eco-label literature by shifting attention from the presence of eco-labels to the credibility of eco-labels as a psychological mechanism underlying sustainable consumption decisions. Existing research has primarily examined whether eco-labels encourage environmentally responsible purchasing by serving as sustainability cues [3,5]. While these studies have established the effectiveness of eco-labels as market signals, considerably less attention has been devoted to explaining why some eco-labels become persuasive whereas others fail to influence consumer decisions. By demonstrating that eco-label credibility influences sustainable consumption intention through consumers’ cognitive evaluations rather than through simple label exposure, this study extends signaling research from the existence of sustainability signals to consumers’ evaluation of signal credibility. The findings suggest that the effectiveness of eco-labels depends not merely on whether sustainability information is provided, but on whether that information is perceived as sufficiently credible to support subsequent cognitive judgments.

Second, this study extends consumer information processing theory by demonstrating that credible sustainability signals influence sustainable consumption through two distinct cognitive mechanisms. Drawing upon Bettman’s [37] information processing theory, Payne et al.’s [38] adaptive decision-making framework, and Woodruff’s [42] customer value perspective, this research distinguishes between an information interpretation pathway and a benefit evaluation pathway. Specifically, eco-label credibility enhances consumers’ perceived diagnosticity by improving the decision usefulness of sustainability information, while simultaneously increasing perceived value by strengthening evaluations of the benefits associated with eco-labeled products. Although previous studies have frequently examined perceived diagnosticity or perceived value independently, the present findings show that these two mechanisms represent complementary cognitive processes rather than competing explanations. This integrated perspective provides a more comprehensive account of how credible sustainability information is translated into sustainable consumption intention and enriches the application of consumer information processing theory in digital retail environments.

Third, this study contributes to research on boundary conditions in sustainable consumption by demonstrating that different cognitive evaluation mechanisms exhibit different levels of sensitivity to economic considerations. Specifically, price sensitivity significantly weakens the positive influence of perceived diagnosticity on sustainable consumption intention, whereas no comparable moderating effect was observed for perceived value. Rather than suggesting that price considerations uniformly constrain sustainable purchasing decisions, these findings indicate that economic concerns primarily influence the translation of information-based judgments into behavioral intention. In contrast, the relationship between perceived value and sustainable consumption intention remained relatively stable across different levels of price sensitivity in the present sample. Together, these findings contribute to a more nuanced understanding of how economic considerations interact with different stages of consumers’ cognitive evaluation processes, highlighting that the boundary conditions of sustainable consumption may vary across distinct psychological mechanisms rather than operating uniformly throughout the decision-making process.

### 6.2. Practical Implications

The findings of this study provide several practical implications for digital retail platforms that integrate eco-labels into online grocery shopping environments, particularly platform-based retailers such as Hema Fresh, where consumers make rapid purchasing decisions based on digitally presented sustainability information.

First, platform managers should prioritize enhancing the credibility of eco-label information rather than simply increasing the number of sustainability labels displayed on product pages. The findings demonstrate that eco-label credibility serves as the starting point for both consumers’ information interpretation and value evaluation processes. For Hema Fresh, this suggests that sustainability labels should be accompanied by easily accessible certification details, issuing organizations, and verification standards within the product interface. Instead of treating eco-labels as static visual symbols, the platform should design product pages that allow consumers to verify why a product has obtained a particular environmental certification, thereby strengthening the credibility of sustainability claims before consumers begin evaluating product information.

Second, digital retail platforms should improve the interpretability of sustainability information rather than merely providing additional environmental content. Because consumers’ sustainable purchase intentions are strengthened when sustainability information is perceived as more diagnostic, the presentation of environmental information should facilitate efficient decision-making instead of increasing informational complexity. For example, Hema Fresh could reorganize sustainability-related information into concise, decision-oriented modules that clearly communicate the environmental attributes, certification basis, and practical implications of eco-labeled products. Such interface optimization would enable consumers to more easily distinguish certified products from conventional alternatives during online shopping.

Third, the significant moderating role of traceability suggests that eco-labels and traceability systems should be designed as complementary platform functions rather than independent features. Hema Fresh already provides supply-chain information for many fresh food products, yet the findings indicate that the managerial value of traceability extends beyond operational transparency. By integrating traceability records more closely with eco-label information—for example, allowing consumers to directly access certification evidence, production records, logistics information, and inspection histories through a unified product interface—the platform may further reinforce consumers’ confidence in the credibility of sustainability claims and strengthen the effectiveness of eco-labels in shaping cognitive evaluations.

Finally, the findings indicate that pricing strategies should differ according to the stage of consumers’ decision-making process. Because price sensitivity weakens the influence of perceived diagnosticity on sustainable consumption intention, price-sensitive consumers may require additional decision support after they have recognized the usefulness of sustainability information. For Hema Fresh, this suggests that temporary member discounts, bundled promotions for eco-labeled products, or personalized recommendations highlighting environmentally certified products within comparable price ranges may be more effective than simply providing additional sustainability information. At the same time, because no moderating effect of price sensitivity was observed for the relationship between perceived value and sustainable consumption intention, managers should continue investing in strategies that enhance consumers’ perceptions of the overall benefits associated with eco-labeled products, including product quality, freshness, environmental performance, and health-related attributes.

## 7. Limitations and Future Research Directions

This study has several limitations that also provide specific directions for future research. First, the study focused on a single digital retail platform, Hema Fresh, within the Chinese online grocery context. Platform characteristics, eco-label systems, and traceability practices may differ across retailers and product categories, potentially affecting how consumers interpret sustainability information. Future research could replicate the proposed model across multiple digital grocery platforms and compare product categories with different levels of sustainability relevance, such as fresh produce, organic food, and packaged food. Cross-cultural studies could further examine whether the effects of eco-label credibility and traceability remain consistent across markets characterized by different sustainability regulations, labeling systems, and consumer familiarity with environmental claims. Such comparative designs would help identify the contextual boundaries of the dual cognitive pathways proposed in this study.

Second, the study relied on a non-probability convenience sample recruited primarily through Wenjuanxing and the research team’s social media networks. Although the final sample was adequate for estimating the proposed structural model, it should not be regarded as statistically representative of the broader population of Hema Fresh users or Chinese online grocery consumers. The recruitment procedure may also have introduced self-selection and channel-related biases, while several sociodemographic groups were unevenly represented. In particular, respondents aged over 55 and those with postgraduate or doctoral education constituted relatively small proportions of the sample. Future studies could employ stratified or probability-based sampling procedures to achieve more balanced representation across age, education, income, and other relevant demographic groups. Multi-group analyses could then examine whether the proposed relationships differ systematically across consumer segments, particularly between younger and older consumers or across different educational and income groups.

Third, the cross-sectional survey design limits causal inference regarding the relationships among eco-label credibility, perceived diagnosticity, perceived value, and sustainable consumption intention. Future research could employ controlled experiments that manipulate eco-label credibility and traceability information independently. For example, participants could be exposed to high- versus low-credibility eco-label information combined with high- versus low-traceability product information, allowing researchers to test their independent and interactive causal effects on perceived diagnosticity, perceived value, and subsequent product choice. Longitudinal studies could complement such experiments by examining whether repeated exposure to credible eco-labels and traceability information produces persistent changes in consumer evaluations and sustainable purchasing intentions over time.

Finally, the present model examined food traceability and price sensitivity as two specific boundary conditions, but the findings suggest several more targeted opportunities for extending the theoretical framework. In particular, the significant moderating effect of price sensitivity on the perceived diagnosticity–intention relationship, together with its nonsignificant moderation of the perceived value–intention relationship, indicates that economic constraints may operate differently across the two cognitive pathways. Future research could investigate why price sensitivity constrains the translation of diagnostic information into sustainable consumption intention but does not significantly alter the value-based pathway. Variables such as environmental knowledge, green trust, skepticism toward sustainability claims, and digital literacy could also be examined as theoretically differentiated boundary conditions rather than simply added to the model. For example, environmental knowledge and digital literacy may influence consumers’ ability to interpret eco-label and traceability information, whereas green trust and sustainability skepticism may affect whether such information is accepted as credible. Examining these mechanisms would help refine the conditions under which credible sustainability information is translated into sustainable food choices in digital retail environments.

## Figures and Tables

**Table 1:** Demographics profile (*n* = 496).

Variable	Category	Frequency	%
Gender	Male	219	44.2
	Female	277	55.8
Age	18–25	116	23.4
	26–35	119	24.0
	36–45	130	26.2
	46–55	104	21.0
	>55	27	5.4
Education	High school and below	83	16.7
	College/Junior college	401	80.8
	Post-graduate	9	1.8
	Doctor and beyond	3	0.6
Salary (Yuan)	<5000	52	10.5
	5000–8000	136	27.4
	8001–10,000	162	32.7
	10,001–15,000	99	20.0
	>15,000	47	9.5

**Table 2:** Reliability and convergent validity of the measurement model.

Variable	Items	Factor Loading	Cronbach’s α	CR	AVE
Eco-label Credibility	ELC1	0.805	0.901	0.901	0.647
	ELC2	0.805			
	ELC3	0.782			
	ELC4	0.809			
	ELC5	0.817			
Perceived Diagnosticity	PD1	0.808	0.891	0.891	0.620
	PD2	0.755			
	PD3	0.794			
	PD4	0.786			
	PD5	0.793			
Perceived Value	PV1	0.819	0.897	0.897	0.635
	PV2	0.809			
	PV3	0.778			
	PV4	0.782			
	PV5	0.797			
Traceability	TR1	0.794	0.895	0.896	0.632
	TR2	0.787			
	TR3	0.813			
	TR4	0.805			
	TR5	0.776			
Price Sensitivity	PS1	0.783	0.879	0.879	0.593
	PS2	0.793			
	PS3	0.777			
	PS4	0.728			
	PS5	0.726			
Sustainable Consumption Intention	SCI1	0.819	0.877	0.877	0.640
	SCI2	0.778			
	SCI3	0.809			
	SCI4	0.794			

(Note. CR = composite reliability; AVE = average variance extracted.)

**Table 3:** Discriminant Validity.

	Mean.	S.D.	Kurtosis	Skewness	ELC	PD	PV	TR	PS	SCI
ELC	4.442	1.388	−0.100	−0.921	0.804					
PD	4.532	1.322	−0.116	−0.739	0.390	0.787				
PV	4.534	1.350	−0.081	−0.904	0.394	0.523	0.797			
TR	4.565	1.339	−0.163	−0.786	0.361	0.449	0.460	0.795		
PS	4.173	1.309	−0.121	−0.898	0.337	0.371	0.337	0.273	0.770	
SCI	4.479	1.393	−0.126	−0.888	0.395	0.492	0.453	0.466	0.332	0.800

**Table 4:** Discriminant validity assessment using the heterotrait–monotrait ratio (HTMT).

	ELC	PD	PV	TR	PS	SCI
ELC						
PD	0.393					
PV	0.394	0.522				
TR	0.367	0.449	0.463			
PS	0.335	0.370	0.335	0.276		
SCI	0.395	0.489	0.455	0.467	0.331	

(Note. HTMT = heterotrait–monotrait ratio. Values below 0.85 indicate satisfactory discriminant validity.)

**Table 5:** Goodness-of-fit indices for the structural equation model.

Classification	X^2^/df	RMSEA	IFI	TLI	CFI	GFI	NFI
Criteria	<3	<0.08	>0.9	>0.9	>0.9	>0.9	>0.9
Value	1.846	0.041	0.977	0.974	0.977	0.947	0.931

(Note. χ^2^/df = chi-square divided by degrees of freedom; RMSEA = root mean square error of approximation; IFI = incremental fit index; TLI = Tucker–Lewis index; CFI = comparative fit index; GFI = goodness-of-fit index; NFI = normed fit index; SRMR = standardized root mean square residual.)

**Table 6:** Structural path estimates and hypothesis testing results.

H	Path			β	S.E.	C.R.	*p*	Supported
H1	PD	<---	ELC	0.415	0.049	8.271	***	Yes
H2	PV	<---	ELC	0.417	0.051	8.343	***	Yes
H3	SCI	<---	PD	0.372	0.050	7.567	***	Yes
H5	SCI	<---	PV	0.295	0.046	6.157	***	Yes

(Note. β = standardized path coefficient; S.E. = standard error; C.R. = critical ratio; *p* = significance level. *** *p* < 0.001.)

**Table 7:** Mediation effect.

Path	Estimate	SE	95%CI		Result
			Lower	Upper	
ELC→PD→SCI	0.154	0.030	0.099	0.215	Support
ELC→PV→SCI	0.123	0.026	0.075	0.179	Support

## Data Availability

The original contributions presented in this study are included in the article. Further inquiries can be directed to the corresponding author.

## Appendix A

**Table A1:** Questionnaire items.

Variable	Items	Question	Reference
Eco-label Credibility	ELC1	I believe that the information provided by this eco-label is truthful and credible.	[5,8,9]
	ELC2	I trust that the certification standards behind this eco-label are reliable.	
	ELC3	I believe that the organization issuing this eco-label has sufficient expertise and credibility.	
	ELC4	I trust that products carrying this eco-label truly meet the environmental standards they claim.	
	ELC5	Overall, I consider this eco-label to be trustworthy.	
Perceived Diagnosticity	PD1	The eco-label helps me clearly judge whether the product is truly environmentally friendly.	[10,25,26]
	PD2	The eco-label provides useful information that helps me make purchase decisions.	
	PD3	The eco-label makes it easier for me to differentiate the environmental performance of different products.	
	PD4	Compared with other sources of information, the eco-label offers a more reliable basis for evaluation.	
	PD5	The eco-label increases my confidence in evaluating the sustainability attributes of the product.	
Perceived Value	PV1	The eco-label makes me feel that the product offers high overall value.	[5,11,31]
	PV2	The environmental benefits indicated by the eco-label make the product worth purchasing.	
	PV3	Given its price, the eco-friendly features signaled by the eco-label make it good value for money.	
	PV4	The eco-label makes the product seem superior to alternatives without such labels.	
	PV5	Using products with eco-labels provides me with a sense of satisfaction.	
Traceability	TR1	The eco-label provides clear information about the origin of the product, making it traceable.	[12,48,54]
	TR2	The eco-label allows me to understand the production or operational process of the product.	
	TR3	The information provided by the eco-label enables me to verify whether its environmental claims are genuine.	
	TR4	The eco-label helps me track key stages of the product along the supply chain.	
	TR5	The eco-label increases my perception of transparency regarding the product.	
Price Sensitivity	PS1	I am very sensitive to price when choosing products with eco-labels.	[5,58,60]
	PS2	If a product with an eco-label is more expensive, my intention to purchase decreases.	
	PS3	Even if eco-labels provide environmental benefits, a high price would still make me hesitate to purchase.	
	PS4	When choosing products, I often prioritize price over ecological advantages.	
	PS5	Without a price advantage, I am less likely to purchase products with eco-labels.	
Sustainable Consumption Intention	SCI1	I am willing to choose environmentally sustainable products or services in the future.	[22,40,72]
	SCI2	Even if it requires additional time or effort, I am still willing to choose environmentally friendly products.	
	SCI3	I will prioritize products or services with lower environmental impact in my future consumption decisions.	
	SCI4	I am willing to continuously support businesses or brands that are committed to environmental sustainability.	
